# Effect of Sub-Inhibitory Concentrations of Nitrofurantoin, Ciprofloxacin, and Trimethoprim on In Vitro Biofilm Formation in Uropathogenic *Escherichia coli* (UPEC)

**DOI:** 10.3390/medsci11010001

**Published:** 2022-12-20

**Authors:** Shane Whelan, Mary Claire O’Grady, Gerard Daniel Corcoran, Karen Finn, Brigid Lucey

**Affiliations:** 1 Department of Biological Sciences, Munster Technological University, Bishopstown, T12 P928 Cork, Ireland; 2 Department of Clinical Microbiology, Cork University Hospital, Wilton, T12 DC4A2 Cork, Ireland; 3 Department of Analytical, Biopharmaceutical and Medical Sciences, Atlantic Technological University, ATU Galway City, Dublin Road, H91 T8NW Galway, Ireland

**Keywords:** uropathogenic *E. coli*, biofilm formation, sub-inhibitory antibiotics

## Abstract

The purpose of this study was to determine the effect of sublethal concentrations of nitrofurantoin, ciprofloxacin, and trimethoprim on biofilm formation in 57 uropathogenic *Escherichia coli* strains (UPEC). The minimum inhibitory concentration of nitrofurantoin, ciprofloxacin, and trimethoprim was determined and the biofilm formation for each isolate with and without sub-lethal concentrations of each antibiotic was then quantified. The statistical significance of changes in biofilm formation was ascertained by way of a Dunnett’s test. A total of 22.8% of strains were induced to form stronger biofilms by nitrofurantoin, 12% by ciprofloxacin, and 19% by trimethoprim; conversely 36.8% of strains had inhibited biofilm formation with nitrofurantoin, 52.6% with ciprofloxacin, and 38.5% with trimethoprim. A key finding was that even in cases where the isolate was resistant to an antibiotic as defined by EUCAST, many were induced to form a stronger biofilm when grown with sub-MIC concentrations of antibiotics, especially trimethoprim, where six of the 22 trimethoprim resistant strains were induced to form stronger biofilms. These findings suggest that the use of empirical treatment with trimethoprim without first establishing susceptibility may in fact potentiate infection in cases where a patient who is suffering from a urinary tract infection (UTI) caused by trimethoprim resistant UPEC is administered trimethoprim. This emphasizes the need for laboratory-guided treatment of UTI.

## 1. Introduction

Urinary tract infections (UTIs), both uncomplicated and complicated, are one of the most common hospital and community associated infections, with a prevalence of 4% of total health care associated infections in the United States and 6% of health care associated infections in Europe [1]. Women are more affected than men, with an estimated 40% of women experiencing a UTI at least once in their lifetime [2]. Additionally, 20–30% of women with UTI will experience a recurrence within 4 months of initial infection [3]. *Escherichia coli* (*E. coli*) is the most common causative organism, accounting for 74.4% of outpatient infections and 65% of hospital acquired infections, and UTI caused by *E. coli* is the most common source of *E. coli* bacteremia [4]. The annual primary care costs to treat UTIs in Ireland were estimated as EUR 19.2 million annually in 2014 [5].

The European Association of Urology (EAU) defines uncomplicated UTI as acute sporadic or recurrent lower UTI, which is limited to non-pregnant women with no relevant anatomical or functional abnormalities within the urinary tract, while a UTI is defined as complicated if the patient is male, a pregnant woman, or has a relevant anatomical or functional abnormality or any relevant comorbidity including renal disease and diabetes, which may complicate treatment [6]. The EAU recommends the following for the treatment of uncomplicated UTI: nitrofurantoin and fosfomycin are recommended as a frontline treatment, as resistance to nitrofurantoin remains comparatively low. Trimethoprim-sulfamethoxazole is to be used empirically in cases where the local resistance rates are <20%, and fluoroquinolones such as ciprofloxacin are recommended as a second-line empiric therapy in cases of mild or moderate pyelonephritis or complicated UTI [6]. Local guidelines recommend that nitrofurantoin or cephalexin be used for uncomplicated UTIs after a positive microbiological test, and trimethoprim is used empirically.

Within the urinary tract and bladder, bacteria can aggregate into biofilms, forming reservoirs of infection that are difficult to eradicate even with antibiotic concentrations that far exceed the minimum inhibitory concentration [7], and the ability of a uropathogenic *Escherichia coli* (UPEC) isolate to form biofilms has previously been shown to be associated with recurrent infection [8]. Bacteria, which are exposed to concentrations of antibiotics that are below the minimum inhibitory concentration (MIC), have been shown to exhibit changes in gene expression [9,10]. Sub-MIC concentrations of ciprofloxacin have been reported to increase biofilm formation in *E. coli* in some studies [11], while in others, it has been reported to reduce biofilm formation [12]. The effect of sub-MIC trimethoprim and nitrofurantoin on *E. coli* biofilm formation have, to our knowledge, not been widely reported. The frequency of biofilm formation in trimethoprim/sulfamethoxazole resistant UPEC has been reported to be significantly higher than in susceptible strains [13], however, and nitrofurantoin has previously been reported to stimulate biofilm production in *Pseudomonas aeruginosa* [14].

The purpose of the current study was to determine the effect of sub-inhibitory concentrations of nitrofurantoin, trimethoprim, and ciprofloxacin on the biofilm forming abilities of 57 isolates of UPEC.

## 2. Materials and Methods

A total of 57 UPEC isolates, each being the causative organism in an individual case of UTI and identified to the species level using MALDI–TOF by Cork University Hospital, were investigated in this study. *E. coli* ATCC 25922 was used as a positive control for antimicrobial susceptibility testing as well as biofilm formation, the selection of the latter being based on the findings of Naves et al. (2008) [15]. *E. coli* K12 MG1655 was also included as it has previously been described as positive for biofilm formation [16].

The susceptibility of UPEC isolates to nitrofurantoin, ciprofloxacin, and trimethoprim was determined using the broth microdilution method, as previously described [17]. The wells of a 96 well microtiter plate were filled with 200 μL of Mueller Hinton broth, diluted with antibiotic solution to make up specified concentrations for each antibiotic. Concentrations were chosen according to the European Committee on Antimicrobial Susceptibility Testing (EUCAST) guidelines regarding the breakpoint values for the minimum inhibitory concentrations for each antibiotic, so that susceptibility could be determined [18].

Briefly, nitrofurantoin was added to the broth to reach final concentrations of 65, 32.5, 25, 12.5, 6.25, 3.125, 1.56, 0.78, 0.39, and 0 mg/L. Ciprofloxacin was added to the broth to reach concentrations of 0.313, 0.157, 0.078, 0.039, 0.02, 0.01, 0.005, and 0 mg/L. Trimethoprim was added to the broth to reach concentrations of 1.25, 0.625, 0.313, 0.157, 0.078, 0.039, 0.02, and 0 mg/L. Concentrations were chosen so that EUCAST cutoff values were encompassed [18]. All concentrations were made so that the final volume of each well was 200 μL. A triplicate of wells for each concentration was inoculated using a 1 μL inoculating loop with a 0.5 MacFarland suspension of each UPEC isolate. The plates were then incubated at 37 °C for 20–24 h without agitation, after which the plates were examined for visible turbidity at each concentration. Absorbance was read using an automatic plate spectrophotometer at 600 nm. Concentrations that resulted in the visual confirmation of non-turbid plate wells combined with an OD600 nm reading below 0.100 were considered above the MIC for each antibiotic.

Following the determination of the MIC for each isolate for each antibiotic, wells that contained sub-MIC concentrations of each antibiotic were analyzed for biofilm formation. Unattached cells and media were aspirated from the plates following MIC determination, and wells were washed three times with 200 μL of 0.8% sterile saline solution. Biomass was heat fixed by placing plates in an 80 °C oven for 1 h. Attached biomass was then stained with 0.5% crystal violet (CV) and incubated at room temperature for 10 min. Unattached CV was aspirated, and wells were washed three times with 200 μL of 0.8% sterile saline solution. A volume of 210 μL of 30% acetic acid was added to each well to solubilize the attached CV. The plates were read spectrophotometrically at 590 nm to determine the related absorbance values. Strains were considered positive for biofilm formation if they formed biofilms resulting in an absorbance reading at least 90% of the biofilm positive control *E. coli* ATCC 25922. The biofilm formation of each strain (with no antibiotics added) was then compared to their biofilm formation at each sub-inhibitory concentration.

The absorbance values at 590 nm were averaged from the triplicate of wells at each concentration. These averages were compared to the average absorbance values of the control conditions (no antibiotic added) by way of a Dunnett’s test using R Studio [19] to determine the significance (*p* ≤ 0.05). Strains were considered induced if they produced stronger biofilms at any sub-inhibitory concentration and the difference was found to be statistically significant. Strains that showed a significant decrease in biofilm formation at any sub-inhibitory concentration were considered reduced and strains that were not significantly induced or reduced were considered unaffected.

## 3. Results

### 3.1. Susceptibility of UPEC to Antimicrobials

Three UPEC strains (5.3%) were found to be resistant (above the EUCAST-defined clinical breakpoint) to nitrofurantoin, 13 (22.8%) UPEC were resistant to ciprofloxacin, and 22 (38.6%) were resistant to trimethoprim. Multidrug resistance was common, with three strains (5.3%) exhibiting resistance to all three antibiotics and 11 strains (19.3%) showing resistance to ciprofloxacin and trimethoprim.

### 3.2. The Effect of Sub-Inhibitory Concentrations of Antibiotics on Biofilm Formation

Biofilm formation was quantified at concentrations below the MIC and compared to biofilm formation in the absence of antibiotics, and the results are shown in Table 1. By way of explanation, an identifying code was created to show this information, whereby the letters N, C, and T indicate that the effect was observed when exposed to subinhibitory concentrations of nitrofurantoin, ciprofloxacin, or trimethoprim, respectively. The ↑ symbol indicates that increased biofilm formation was found when compared to growth with no antibiotic, while the ↓ indicates that a reduction in biofilm formation was seen. The strains were also grouped based on their resistance to trimethoprim, ciprofloxacin, and nitrofurantoin.

**Figure 1:**
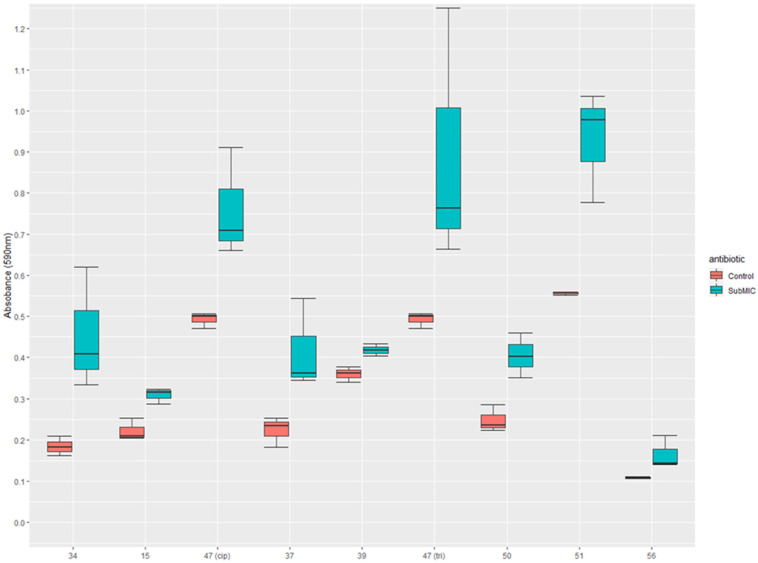
Induction of biofilm formation by sub-inhibitory concentrations of either nitrofurantoin, ciprofloxacin, or trimethoprim for nine resistant UPEC strains whereby the results are shown in comparison to a control for that strain without the antibiotic present.

Strains that showed significantly enhanced biofilm formation when exposed to sub-inhibitory concentrations of an antibiotic to which they were also resistant are presented in a boxplot (Figure 1). This shows the variation between replicates (visible by the upper and lower error bars) and the median of triplicates. Strains 15 and 47 are strains induced by sub-inhibitory ciprofloxacin at concentrations of 0.005 mg/L and 0.078 mg/L, respectively, when compared to the control with no antibiotic. Strain 15 produced 28% more biofilm and strain 47 produced 35% more biofilm when grown with sub-inhibitory ciprofloxacin. Strain 34 showed biofilm formation with 25 mg/L of nitrofurantoin and produced 59% more biofilm than when compared to the control. Samples 37, 39, 47, 50, 51, and 56 showed the induction of biofilm formation by trimethoprim at 1.25 mg/L. These strains showed increases of 46%, 14%, 44%, 38%, 40%, 34%, and 25%, respectively. Strain 47 was resistant to both ciprofloxacin and trimethoprim and was induced to form stronger biofilms by both ciprofloxacin and trimethoprim and was labelled as 47(cip) and 47(tri) to reflect this.

Figure 1 shows the differences in biofilm formation between resistant strains that were significantly induced (*p* ≤ 0.05) to form stronger biofilms in vitro when incubated with sub-inhibitory concentrations of antibiotics. Significance was determined using a Dunnett’s test. The *p*-values for each comparison are as follows; 34: *p* = 0.0011, 15: *p* = 0.0071, 47(cip): *p* = 0.0011, 37: *p* = 0.00049, 39: *p* = 0.0102, 47(tri): *p* = 0.005, 50: *p* = 0.00047, 51: *p* = 0.00067, 56: *p* = 0.0427.

## 4. Discussion

In this study, we report the antibiotic susceptibility of 57 UPEC isolated from patients with urinary tract infection in Cork, Ireland for the purposes of studying the effect of sublethal doses of antibiotics on biofilm formation. Two of the 57 (3.5%) strains were resistant to nitrofurantoin, 13 of 57 (22.8%) strains were resistant to ciprofloxacin, and 22 of 57 (38.6%) strains were resistant to trimethoprim. Fourteen strains were resistant to more than one antibiotic (24.5%), with 11 (19.3%) of those being resistant to ciprofloxacin and trimethoprim only; two (3.5%) strains were found to be resistant to all three antibiotics. Published reports on antibiotic susceptibility patterns show that the susceptibility rates vary by region, and resistance is higher in the developing world [20]. An analysis of susceptibility test results of Enterobacteriaceae to antibiotics in the Cork region of Ireland in 2018 determined a resistance rate of 12.8% for ciprofloxacin, 8.5% for nitrofurantoin, and 30.8% for trimethoprim (n = 8999) [21]. The susceptibility results we present in this study show the resistance rates for this region when focused specifically on *E. coli* and show a notably higher rate of resistance to ciprofloxacin and trimethoprim. As previously stated, the EUA recommends fosfomycin as a front-line treatment for UTI. While resistance rates to fosfomycin remain low, recent reports indicate that the fosfomycin susceptibility rates are lower among ESBL producing *E. coli* due to the *fosA3* gene commonly found on the same conjugative plasmid as ESBL-encoding genes [22]. None of the UPEC investigated in this report were found to be resistant to fosfomycin, when susceptibility to this antibiotic was checked.

Biofilm formation was variable, with 46 of the 57 (80.7%) strains being positive for biofilm formation and 11 strains (19.3%) being weak or non-biofilm formers. No relationship was found between biofilm formation and resistance; there was no significant difference in the mean biofilm formation of the population of UPEC that were resistant to any antibiotic when compared to the population of UPEC that were susceptible to all three antibiotics. This was confirmed by a pairwise t-test between these two groups (*p* ≥ 0.05). These findings are in agreement with those of others [23,24], that is, biofilm formation for resistant strains is variable, and not all were strong biofilm formers. Biofilm formation in uropathogens cause difficulty in treatment, as biofilms provide an intrinsic resistance to antimicrobials [25]. The minimum biofilm eradicating concentration (MBEC) is generally much higher than the MIC for a given antibiotic [26], additionally, biofilms may assist with the colonization of the bladder, forming a reservoir of cells that persist through antibiotic intervention and lead to recurrence [7]. Wang et al. (2020) investigated the *E. coli* biofilm eradication efficacy power of trimethoprim, nitrofurantoin, and ciprofloxacin at high concentrations, measured with resonant hyperspectral imaging. They found that even at very high concentrations (500× MIC), trimethoprim and nitrofurantoin were ineffective at eradicating an established biofilm. However, they did find that ciprofloxacin was effective at 300× the MIC. This may be attributable to ciprofloxacin’s ability to inhibit DNA replication [27].

It has been suggested that the sub-lethal doses that most microbial communities are naturally exposed to play a role in cell-to-cell signaling and communication [28]. In laboratory settings, exposing bacteria to sub-inhibitory concentrations of antibiotics has been demonstrated to have a variety of effects including many that may potentially increase virulence [10,11,29]. Our study shows that the effect of sub-inhibitory concentrations of antibiotics on biofilm formation are strain dependent (Table 1).

Sub-MIC ciprofloxacin-mediated biofilm enhancement in UPEC has previously been reported by Rafaque et al. (2020), which showed that five out of six UPEC isolates in that study were induced to form stronger biofilms with sub-MIC ciprofloxacin [11]. Inhibition by ciprofloxacin in *E. coli* including ATCC 25922 has also been reported previously by Dong et al. (2019), who showed that *fim* genes as well as the pgaABCD locus were suppressed at one quarter the MIC; for ATCC 25922, this was 0.00375 mg/L [12]. Importantly, the pgaABCD locus promotes polysaccharide synthesis [30], while *fim* encodes for the type 1 pilus, which are important for mediating initial attachment [31]. Some of the other most important virulence genes for the formation of *E. coli* biofilms are the *pap* and *sfa* genes. The *sfa* gene was found to be expressed strongly in strong biofilm forming UPEC, while the *afa* gene was found to be expressed strongly in weak biofilm formers [32]. The *sfa* operon encodes mannose-resistant adhesions, allowing adhesion to mucosal, endothelial cells, and tissue matrices [33]. Sub-inhibitory concentrations of both nitrofurantoin and ciprofloxacin have previously been shown to upregulate genes encoding S fimbrial adhesion (sfa) and FiC fimbriae (foc) proteins in UPEC [10]. Ciprofloxacin has also been shown to increase the hemagglutination titer and surface hydrophobicity of UPEC strains, resulting in a higher expression of surface proteins, which can increase adhesiveness [29].

Notable for this study is that many resistant strains were affected by sub-inhibitory concentrations of antibiotics, with eight resistant strains being induced to form stronger biofilms when exposed to sub-MIC nitrofurantoin, ciprofloxacin, or trimethoprim (Figure 1). One of two nitrofurantoin resistant strains exhibited upregulated biofilm formation when exposed to a sub-lethal dose of antibiotic, resulting in a biomass increase of 59% when grown with 25 mg/L nitrofurantoin. Ciprofloxacin enhanced biofilm formation in two ciprofloxacin resistant strains caused a biofilm increase of 38.9% in strain 15 and an increase of 54.2% in strain 47 at 0.005 mg/L and 0.078 mg/L, respectively. Trimethoprim had the highest number of resistant strains that were also induced to form the biofilm by sub-MIC trimethoprim, with samples 37, 39, 47, 50, 51, and 56 showing enhanced biofilm formation with trimethoprim at 1.25 mg/L. Strain 47 is notable here as it was found to be resistant to both trimethoprim and ciprofloxacin, and biofilm formation was induced by both antibiotics. This is especially relevant as trimethoprim is often used empirically, without prior susceptibility testing. In this case, it is possible that intervention may unknowingly exacerbate an infection, even when treated subsequently with an antibiotic to which the organism is susceptible. A limitation of this study was the sample size of UPEC investigated and the true prevalence of sub-MIC trimethoprim mediated biofilm enhancement may not be fully elucidated until a larger study is carried out. A multivariate analysis of college age women in the United States who were prescribed antibiotics for a UTI found that prescription with trimethoprim-sulfamethoxazole was significantly associated with reoccurrence of infection [34], which may be explained by the findings in this study.

## 5. Conclusions

We demonstrate that sub-inhibitory concentrations of antibiotics can enhance biofilm formation, both among EUCAST-defined susceptible or resistant strains. The combination of frequent trimethoprim resistance among UPEC and this antibiotic’s propensity to induce or upregulate biofilm formation suggests that empirical use of this antibiotic for UTI may mitigate against future effective treatment of UTI caused by these strains.

## Figures and Tables

**Table 1:** The effect of sub-inhibitory concentrations of nitrofurantoin, ciprofloxacin, and trimethoprim on biofilm formation in UPEC.

Resistance	Effect on Biofilm Formation	Strains in Phenotype
Resistant to none	None	1, 5, 18, 23, 58, ATCC 25922
	N↓ C↓ T↓	20, 41, 43, 46, 55
	C↓ T↓	3, 10, 12, 52
	N↑ C↑	2, 60
	N↓ T↓	45, MG1655 K12
	C↓	13, 59
	T↓	7, 8
	N↑ T↑ C↓	4, 36
	N↓	27
	N↓ C↓ T↑	29
	N↑ C↑ T↑	38
	N↓ C↑ T↓	40
	N↑ C↓ T↓	62
	N↓ T↑	28
	N↓ C↑	31
	N↑ C↓	61
Trimethoprim	N↓ C↓ T↓	11, 22, 30
	N↑ C↓ T↑	50
	N↓ T↑	39
	N↓ C↓	44
	C↓ T↑	51
	C↓ T↓	53
	N↑ T↑	37
	N↓	33
	N↑	9
	C↓	16
	None	32
Ciprofloxacin	N↓ C↓ T↓	42
	C↑	15
	N↓	24
	None	25
Ciprofloxacin and Trimethoprim	N↓ C↓ T↓	35, 54
	None	17, 57
	N↑ T↑	47
	C↑ T↑	56
	C↓	48
Resistant to all	N↓ C↓ T↓	49
	N↑ C↓	34

Table 1 shows the 57 UPEC isolates and *E. coli* strains ATCC 25922 and K12 MG1655 separated into phenotypic groups based on the results of the antimicrobial susceptibility testing and the effect of subinhibitory concentrations of nitrofurantoin, ciprofloxacin, and trimethoprim on biofilm formation.

## Data Availability

The data presented in this study are available in Supplementary Tables S1 and S2.

## Supplementary Materials

The following supporting information can be downloaded at: https://www.mdpi.com/article/10.3390/medsci11010001/s1, Table S1: Absorbance readings at 590 nm following biofilm staining protocol of UPEC isolates grown with varying concentrations of antibiotics; Table S2: The minimum inhibitory concentration (MIC) of nitrofurantoin, ciprofloxacin, and trimethoprim for each UPEC isolate.
